# Evidence on sexual and reproductive health service delivery during and post COVID-19: a multi-country facility assessment

**DOI:** 10.1186/s12978-025-02185-w

**Published:** 2025-11-20

**Authors:** Moazzam Ali, Grace Kapustianyk, Armando Humberto Seuc, WHO HRP Social Science Research Team, Luis Bahamondes, Jose Guilherme Cecatti, Vilma Zotareli, Rachel E. Soeiro, Karayna G. Fernandes, Mariana B. Rogerio, Samira M. Haddad, Silvana F. Bento, Karla S. Padua, Aline Munezero, Charles M. Charles, Montas Laporte, Eunice Chomi, Seni Kouanda, Flore Marie Gisèle Donessouné, Armel Sogo, Kun Tang, Yueping Guo, Hanxiyue Zhang, Yifan Zhu, Ge Yang, Chunxiao Peng, Xizhuo Xie, Hao Wang, Deda Ogum Alangea, Kwasi Torpey, Emefa Judith Modey, Adom Manu, Ernest T. Maya, Rozina Karmaliani, Laila Ladak, Arusa Lakhani, Marina Baig, Yasmin Parpio, Salima Somani, Pisake Lumbiganon, Jen Sothornwit, Nampet Jampathong, Somporn Rungreangkulkij, Marleen Temmerman, Ferdinand Okwaro, Abdu Mohiddin, Massimo Mirandola, Maddalena Cordioli, Alessia Savoldi, Simone Garzon, Stefano Uccella, Ranieri Poli, Nigel Sherriff, Alexandra Sawyer, Catherine Aicken, Jörg W. Huber, Jaime Vera, Deborah Williams, Caron Kim, Vanessa Brizuela, Hamsadvani Kuganantham, Igor Toskin, Soe Soe Thwin, Anna Thorson, Joy Jerop Chebet, Hugo Gamerro Abrego, Gabriela Garcia Camacho

**Affiliations:** 1 WHO Department of Sexual and Reproductive Health and Research, World Health Organization, Geneva, Switzerland; 2 Instituto Nacional de HigieneEpidemiología y Microbiología, Infanta, 1158 E/Clavel y Llinas, Centro Habana, La Habana, Cuba

**Keywords:** Sexual and reproductive health, COVID-19 pandemic, Health systems resilience, Service disruption, Mixed-methods, Multi-country study

## Abstract

**Introduction:**

The COVID-19 pandemic significantly disrupted global health systems, and sexual and reproductive health (SRH) were among the services often receiving less priority compared to other urgent health needs. Anticipated disruptions included limited access to contraception, abortion care, sexually transmitted infections (STI) prevention, and gender-based violence (GBV) support, particularly in low- and middle-income countries. This study aimed to assess the availability, readiness, and recovery of SRH services during and after the pandemic across nine countries.

**Methods:**

This repeated cross-sectional, mixed-methods study was implemented in collaboration with national Ministries of Health across nine countries: Brazil, Burkina Faso, China, England, Ghana, Italy, Kenya, Pakistan, and Thailand. Data were collected from health facilities and SRH clients at two time points: during peak COVID-19 transmission and post-pandemic. Standardized facility assessments and client interviews were analyzed using multilevel logistic regression models. Qualitative data were collected through in-depth interviews and focus group discussions in selected countries to explore service disruptions, health system adaptations, and psychosocial impacts. Thematic analysis identified context-specific challenges and coping strategies.

**Results:**

Of 4,346 clients interviewed, 2,292 from seven countries were included in the final analysis. Post-pandemic improvements were modest, with gains in STI testing and GBV response training. Family planning and abortion services showed minimal recovery. Systemic vulnerabilities such as outpatient closures, staff shortages, and supply chain failures contributed to service disruptions. Facilities adopted resilience strategies including telemedicine, task shifting, and triaging. Qualitative findings revealed emotional strain among healthcare providers, stigma, and informal coping mechanisms, underscoring the need for integrated mental health support and flexible service delivery models.

**Conclusion:**

The pandemic exposed critical weaknesses in SRH service delivery but also highlighted opportunities for strengthening health system resilience.

Integrating qualitative insights enhances understanding of lived experiences and local innovations, essential for building adaptable and accessible SRH systems. Sustained investment in workforce support, supply chains, and context-specific service models is vital for future health emergency preparedness.

**Supplementary Information:**

The online version contains supplementary material available at 10.1186/s12978-025-02185-w.

## Background

### The onset of COVID-19 and its anticipated impacts on SRH

The severe acute respiratory syndrome coronavirus 2 (SARS-CoV-2), or the 2019 novel coronavirus, was first reported in December 2019. Rapidly spreading globally, it was characterized as a pandemic by the World Health Organization (WHO) in March 2020. Over the last four years, the COVID-19 pandemic has affected all countries in some way. As of April 6, 2025, the total number of COVID-19 cases reported to WHO is 777,704,325. Countries with the highest number of reported cases include the United States of America (103 M), China (99.4 M), India (45 M), and France (39 M) [1].

Total deaths as of April 6, 2025, are 7,093,786, with the United States of America (1.2 M), Brazil (703 K), and India (534 K) having the highest numbers reported. Since the first outbreaks and the declaration of a global pandemic, there have been numerous waves of COVID-19 transmissions throughout 2020, 2021, and 2022, with particularly high counts globally in January 2022. Although smaller in magnitude, waves of case counts continue to persist (e.g., January 2024, April 2024), demonstrating that despite vaccinations and additional health policies, the COVID-19 virus remains present and impacts the global population and its health systems in varying and challenging ways.

The pandemic led to an unprecedented increase in demand on health systems for care for people infected with COVID-19, necessitating the allocation of significant resources towards the response. At its onset, and throughout 2020, many health practitioners, epidemiologists, and infectious disease researchers anticipated that the provision of essential services, including sexual and reproductive health (SRH) services, would be disrupted due to increased demand and movement restrictions [2–4]. This was further supported by previous experiences from the Zika and Ebola outbreaks [5, 6], where some services were unavailable due to facilities and/or health workers being repurposed for care of infected patients. For SRH service disruptions, this meant women and girls were especially exposed to preventable health risks [7]. Several factors might have contributed to SRH service disruption, including:

- Travel to hospitals may be disrupted by movement restrictions to increase the safety of patients and communities [2, 3]
- A reduction in health workers available for service provision because of increasing numbers being themselves infected by COVID-19 [2, 3]
- Clinical staff may not have the time or personal protective equipment needed to provide SRH and family planning counselling and commodities.
- Supply chain disruptions could likely limit the availability of contraceptives in many places.
- Quick mobilization for diagnostic and treatment resources for COVID-19, along with public health measures to contain the spread of infection, could severely disrupt or stop the delivery of SRH-related services altogether.

It was anticipated that significant levels of lockdown-related disruption over six months could leave 47 million women in low- and middle-income countries (LMICs) unable to use modern contraceptives, leading to a projected 7 million additional unintended pregnancies in LMIC [8]. Early pandemic studies modeled the potential impacts, showing that even a 10% reduction in comprehensive SRH services^1^could lead to an estimated 15 million unintended pregnancies, 3.3 million unsafe abortions, and 29,000 additional maternal deaths during the next 12 months [9–12]. The United Nations Population Fund (UNFPA) also estimated an additional 31 million cases of gender-based violence (GBV) due to lockdown measures lasting six months [8, 13]. Maintaining continuity of essential health services while keeping people safe during the response to disease outbreaks such as the COVID-19 pandemic was considered essential to the prevention of both direct and indirect mortality [8, 9]. Early research from the pandemic on COVID-19-related SRH service disruptions found that in-person abortion services were curtailed and access to sexually transmitted infections (STI) and HIV services and GBV services decreased dramatically [14]. Findings from a scoping review on the negative impact of the pandemic on the access to and utilization of SRH services highlighted similar results, and identified transportation disruptions, financial hardships, reduced medical supplies and human resources and access to abortion care as all key barriers to accessing SRH care across the globe [15].

To evaluate the impact of COVID-19 on the health systems' capacity to provide SRH services, the WHO’s Human Reproductive Program (HRP) conducted a multi-country mixed-methods study. Countries with participating institutions included Burkina Faso, Brazil, China, Ghana, Italy, Kenya, Pakistan, Thailand, and the United Kingdom. Data collected provided insights into both regional and global variations in their COVID-19 responses and the ways in which their respective SRH services functioned.

This study consisted of data collected at the individual (service clients and provider) and health facility levels across the nine participating countries with the aim of identifying the key gaps and barriers to the delivery and recovery of important aspects of comprehensive SRH services in health emergencies. This global synthesis assesses contraception, comprehensive abortion- and post-abortion care, STI prevention and treatment, and GBV care availability in local health facilities during the COVID-19 pandemic and post-pandemic. This paper reports on quantitative aspects on the availability and readiness of health facilities to deliver sexual and reproductive health services in regions most affected by COVID-19, in collaboration with the Ministry of Health Additionally, it assesses the post-pandemic recovery of these facilities, comparing their capacity to deliver such services after the pandemic to their performance during the height of the crisis. The synthesis provides a better understanding of extent of these disruptions across the countries at the subnational and facility levels in addition to a set of mitigating strategies employed by each country, which can be applied in any setting, to respond to health emergencies in the future, ensuring that key SRH services are accessible, affordable, and women-centered.

## Methods

### Study design and setting

This study employed a repeated cross-sectional, mixed-methods design the protocol for which has been previously published [16], to assess the availability, readiness, and utilization of sexual and reproductive health (SRH) services during pandemic and post COVID-19 pandemic. This paper provides an overview of the overarching research design and reports on the results. Data were collected at two time points: we defined ‘during pandemic’ as a period of high community transmission (WHO pandemic phases 3–4) and during the ‘post-pandemic’ recovery phase (phases 0–2), approximately 9 to 12 months apart [17].

Research was conducted in selected health facilities across varied geographic locations in nine countries: Brazil, Burkina Faso, China, England, Ghana, Italy, Kenya, Pakistan, and Thailand. This paper examines the availability and readiness of health facilities to deliver sexual and reproductive health (SRH) services in regions most affected by COVID-19. Countries were selected based on expressed interest in conducting the research, and study sites were identified in collaboration with the respective Ministries of Health.

In addition to quantitative assessments, qualitative data were collected through in-depth interviews (IDIs) and focus group discussions (FGDs) with women, partners, and healthcare providers. These methods were used to explore barriers to SRH service access, health system responses, and psychosocial impacts of the pandemic. The qualitative component followed a grounded theory or thematic content analysis approach, depending on country context, and was designed to reach thematic saturation.

### Facility and participant selection

Health facilities were selected based on their capacity to provide a core set of SRH services, including contraception, abortion and postabortion care, STI management, and support for gender-based violence (GBV) survivors. Additional criteria encompassed geographic diversity, urban/rural setting, and the facility's willingness to participate. The final selection aimed to capture a diversity of health system capacities and COVID-19 response structures. The study population included women and their partners seeking SRH services at the selected facilities. Healthcare providers involved included medical doctors, nurses, midwives, and allied health professionals involved in SRH service delivery.

Due to space constraints, this paper also presents some of the qualitative findings from three countries including China, Brazil and Kenya. Detailed results from the remaining participating countries are available in the accompanying supplement. In China, Brazil, and Kenya, purposive sampling was used to recruit participants for the qualitative component. In China, 109 IDIs were conducted across seven facilities in three cities. Brazil included both IDIs and FGDs across three cities, while Kenya focused on IDIs with 40 frontline healthcare workers in 12 facilities. Sampling was guided by data saturation and triangulated across stakeholder groups.

### Data collection and instruments

Data were collected using two internally developed WHO questionnaires. The first was a client questionnaire administered to women attending the facilities. It covered general characteristics, pregnancy status, contraception use, abortion, STI screening, and GBV-related care. A total of 4,346 clients were interviewed. Participants were recruited consecutively across five SRH service areas, and their responses were categorized as "during pandemic" or "post-pandemic" based on country-specific timelines.

The second was a facility assessment questionnaire, completed by one knowledgeable healthcare provider per site. It evaluated clinic performance and readiness across five modules: Health Systems, Family Planning, Abortion services, STIs, and GBV. This tool assessed the availability of policies, trained staff, essential commodities, and infrastructure. Up to 48 clinics completed both during and post pandemic assessments; however, not all facilities responded to every survey module due to pandemic-related logistical constraints, staff shortages, and competing priorities.

For the quantitative analysis, the final sample focused on 2,292 clients from seven countries after excluding Ghana and Burkina Faso due to challenges in defining consistent pandemic periods for comparison. Outcome variables included access to and quality of SRH services, which were analyzed using multilevel logistic regression models adjusted for client and facility characteristics. To capture rich contextual data, qualitative information was gathered through in-depth interviews and focus group discussions with purposively selected women and their partners, aiming for thematic saturation to explore client-reported barriers to accessing care. As stated, these findings are presented in the country-specific papers within this special supplement.

Qualitative data collection was conducted using semi-structured guides co-developed with WHO and adapted to local contexts. Interviews explored service disruptions, barriers to access, health system adaptations (e.g., telemedicine, task shifting), and psychosocial impacts on clients and providers. In Kenya, particular attention was paid to the mental health of healthcare workers, revealing widespread burnout, stigma, and avoidance of COVID-19 testing. In Brazil, FGDs highlighted fear of seeking care and lack of contraceptive prioritization. In China, interviews revealed barriers to SRH access and innovations such as digital counselling and community engagement.

### Sample size and data management

While the study protocol did not specify a formal aggregate sample size calculation at the overall study level, country-specific sample size estimations were conducted. These calculations reflected varying assumptions based on each country’s unique COVID-19 conditions.

Sample size calculations were guided by the World Health Organization’s (WHO) scenario estimations, which were provided to assist study sites. Each participating country team estimated their expected sample size using assumptions about the number of clients seeking sexual and reproductive health (SRH) services per month, adjusted for perceived risks of COVID-19, and considered corresponding margins of error.

Given the unprecedented and unpredictable nature of the pandemic, the study was designed primarily to document service delivery trends and identify gaps, rather than to generate statistically powered comparisons. Consequently, an exact response rate was not calculated. Nevertheless, the design successfully captured essential trends in SRH service delivery under extraordinary circumstances.

Data entry was conducted using electronic databases. WHO-supported centers utilized the OpenClinica online platform.

### Ethical approvals

All participants underwent a comprehensive informed consent process, with written or oral consent obtained as appropriate, particularly in online data collection settings or among participants unable to write. Ethical safeguards, including anonymity, privacy, and confidentiality, were rigorously maintained. Country-specific ethical approvals were secured in addition to overarching approval from the WHO Research Project Review Panel and the WHO Ethics Review Committee. Provisions for referral to local support services were arranged, and data collectors were trained in emotional support and ethical engagement with sensitive topics.

### Data analysis

Descriptive statistics were employed to profile client demographics and facility characteristics. For client-level data, multilevel logistic regression models assessed key SRH service trends during the pandemic and post-pandemic, adjusting for age, education, marital status, pregnancy status, and the primary reason for the visit. These models accounted for clustering effects at the clinic and country levels. Outcome variables were dichotomized for consistency in analysis.

Clinic-level data were evaluated using repeated measurements at these two time points, with all variables presented in binary form. Logistic regression analyses included random effects for country and clinic-level clustering. Cross-tabulations to illustrate directional changes (e.g., from “no” to “yes”) in clinic responses across the study timeline were computed, such that the results are interpreted as changes from during the pandemic to post-pandemic. All analyses were done using R and SPSS v25.0.

Qualitative data were analyzed using thematic content analysis or adapted grounded theory approaches, depending on country context. Transcripts from in-depth interviews and focus group discussions were coded inductively to identify emerging themes. Coding frameworks were developed collaboratively by country teams and refined through iterative review. Data were managed using qualitative analysis software such as Dedoose, and saturation was used to guide sample adequacy. Thematic domains included service disruptions, barriers to access, health system adaptations, and psychosocial impacts on clients and providers.

## Results

### Patient characteristics

Due to multiple waves of the COVID-19 pandemic, only 7 of the 9 participating countries were able to establish clear date cut-offs for the 'during pandemic' and 'post-pandemic' periods. These seven countries are included in the subsequent analysis.^2^ The proportion of patients attending clinics under 20 years increased from 10% during the pandemic to 15.5% post-pandemic, but this change was not statistically significant (*p* = 0.08). The majority of the clients were females, the percentage of male patients decreased from 14.3% to 9.0%, which was also not statistically significant (*p* = 0.5004). For clients seeking care the education levels below secondary remained stable, while the proportion of unmarried or single women visiting health centers slightly increased. The rate of patients tested for COVID-19 significantly increased from 41.2% to 49.3% (*p* = 0.0004). (see Table 1).

**Table 1:** Characteristics of patients

**Background characteristics**	**Pandemic period**						
	**During**			**Post**			*p*-value
	N	n	%	N	n	%	
Age < 20 years	1021	103	10.1%	1271	197	15.5%	0.0876
Sex	1022			1273			
Male		146	14.3		114	9.0	0.5004
Female		876	85.7		1159	91.0	
Education	1022			1274			0.5360
None		69	6.8		37	2.9	
Primary		74	7.2		76	6.0	
Secondary		205	20.1		302	23.7	
High school		339	33.2		447	35.1	
Universisty		282	27.6		346	27.2	
Graduate school		53	5.2		66	5.2	
Marital status	1020			1274			0.2010
Single		272	26.7		349	27.4	
Marital cohabiting		702	68.8		860	67.5	
Separated		28	2.7		36	2.8	
Divorced		15	1.5		24	1.9	
Widowed		3	0.3		5	0.4	
Ethnic group	1017			1271			< 0.001
Han		31	3.0		19	1.5	
Caucasian		108	10.6		103	8.1	
African		333	32.7		410	32.3	
South Asian		145	14.3		107	8.4	
East Asian		2	0.2		2	0.2	
Latino/hispanic		46	4.5		40	3.1	
Middle eastern		5	0.5		3	0.2	
Other		347	34.1		587	46.2	
Tested for COVID-19 (YES)	1022	421	41.2	1273	628	49.3	0.0004

There was a slight decrease in the proportion of currently pregnant patients attending the clinics during pandemic compared to post-pandemic visits, but this was not statistically significant (*p* = 0.2547). Visits related to violence declined significantly over time (*p* = 0.0365), while the rate of induced abortions remained stable. Additionally, over time, contraception care (including counseling and provision) improved modestly but not significantly (*p* = 0.4610). Counseling and screening for STIs showed a small decline, which was not statistically significant (*p* = 0.0715). Testing for STIs other than HIV showed a statistically significant increase (*p* = 0.0684, approaching significance under the study’s exploratory threshold). Concerns about STI testing (*p* = 0.0002) and access to contraception (*p* = 0.0143) significantly increased. Access to STI services and the difficulty in obtaining lab tests and treatments for STIs showed minor changes. The data reveals notable shifts COVID-19 testing rates. The findings emphasize the ongoing need to strengthen healthcare services (see Table 2).

**Table 2:** Reasons or visit and services obtained

	Pandemic period						*p*-value
	During			Post			
	N	n	%	N	n	%	
Currently pregnant	875			1158			0.2547
No		688	78.6		936	80.8	
Yes		187	21.4		222	19.2	
Main reason of visit	1015			1274			0.5876
Post-abortion care		14	1.4		5	0.4	
Pregnancy/antenatal care		145	14.3		176	13.8	
Pregnancy/delivery care		16	1.6		22	1.7	
Postnatal care		103	10.1		155	12.2	
Contraception/FPL		397	39.1		586	46.0	
Help with violence at home		14	1.4		2	0.2	
Screening/treatment for STIs		107	10.5		105	8.2	
Abortion		36	3.5		46	3.6	
Other^#^		183	18.0		177	13.9	
**The following questions were asked to all clients for all main reasons of visits, except "Post-abortion care" and "Abortion":**							
Did anyone ask you about your situation at home, problems with your partner or if you have experienced any form of violence or abuse? (1)	977			1268			0.0365
Yes		128	13.1%		130	10.3%	
Did the client receive family planning/contraception services (including methods) during this current visit? (1)	923			1196			0.4610
Yes		515	55.8%		708	59.2%	
In your lifetime, have you ever been told by a doctor or other provider that you had a sexually transmitted infection (STI), other than HIV? (1)	414			450			0.0684
Yes		146	35.3%		207	46.0%	
At this moment, what do you think are your most important sexual health needs or concerns? (1)	406			451			0.8326
HIV testing		92	22.7%		124	27.5%	
At this moment, what do you think are your most important sexual health needs or concerns? (1)	405			451			0.0002
STI testing		77	19.0%		150	33.3%	
At this moment, what do you think are your most important sexual health needs or concerns? (1)	400			451			0.0143
Access to preferred method of contraception		73	18.3%		136	30.2%	
At this moment, what do you think are your most important sexual health needs or concerns? (1)	400			451			0.1360
Abortion Care		24	6.0%		22	4.9%	
**The following questions were asked all clients whose main reason for the visit was: "Abortion" or”Post-Abortion Care and Abortion Care":**							
Type of care/service for current abortion care visit (2):	49			51			0.9631
Induced abortion		42	85.7		46	90.2	
Did the client receive family planning/contraception services (including method) before hospital discharge? (2)	48			51			0.7940
Counselling and provision		41	85.4%		45	88.2%	
**The following questions were asked to all clients whose main reason for the visit was: "Post-abortion care and abortion care", "Pregnancy/antenatal care", "Screening/treatment for STI", "Abortion", or "Other"**							
Did you receive any screening and/or counseling on STIs? (3)	449			502			0.0715
Yes		142	31.6%		129	25.7%	
**The following questions were asked to all clients for all main reasons of visit, except "Post-abortion care" and "Abortion":**							
Did you have access to the STIs service during the COVID19-related lockdown period? (1)	121			193			0.0584
Yes		57	47.1%		101	52.3%	
HOW DIFFICULT IT WAS: Getting an appointment (1)	1022			1274			0.3450
Difficult		21	2.1%		19	1.5%	
HOW DIFFICULT IT WAS: Getting lab tests for STIs (1)	1022			1274			0.4130
Difficult		19	1.9%		18	1.4%	
HOW DIFFICULT IT WAS: Getting the treatment for STIs (1)	1022			1274			NA
Difficult		13	1.3%		13	1.0%	

*NA* the ML logistic regression did not converge; *p*-value could not be estimated

^*^p-value for fixed effect "Pandemic Period" (During/Post), in Multilevel logistic regression with random effect "Clinic" and "Country". Highlighted in red the option(s) used for dichotomization

^#^ ‘Others’ here refers to clients who came for one or more multiple SRH services and could not be placed in one category, or who’s main reason was not listed in the response options

(1): To be answered by all except those that selected "Post-abortion care and abortion care" or "Abortion", in question "What's the main reason for the current visit"

(2): To be answered only by those that selected "Abortion", "Post-abortion care" or "Abortion care" in question, "What's the main reason for the current visit", as the main reason for current visit

(3): To be answered by those whose main reason for the visit was: "Post-abortion care and abortion care", "Pregnancy/antenatal care", "Screening/treatment for STI"or "Other".

Qualitative data from China, Brazil, and Kenya further contextualized these trends. In China, women reported barriers to accessing SRH services due to lockdowns, lack of information, and limited availability of contraceptive products. In Brazil, fear of infection and unclear communication from health authorities led to reduced service-seeking behavior, particularly for contraception. In Kenya, healthcare providers described widespread burnout, stigma, and avoidance of COVID-19 testing, which impacted service delivery and client-provider interactions. These insights highlight the psychosocial and systemic dimensions of SRH service disruptions beyond what quantitative data alone can capture.

### Health service disruptions and mitigation strategies during the COVID-19 pandemic

Our multi-country analysis revealed disruptions and lack of action across essential health services during the COVID-19 pandemic, with notable variations in service resilience and mitigation approaches. The quantitative findings demonstrate both systemic vulnerabilities and innovative adaptations across seven nations.

### Service disruptions and system vulnerabilities

The analysis of health system functioning during and after the COVID-19 pandemic reveals a largely stable policy and operational environment across the surveyed facilities. During the pandemic, nearly all facilities (42 out of 43) reported that their countries had defined a national SRH essential services package. This number declined to 37 post-pandemic, but the change was not statistically significant (*p* = 0.165). Government funding for essential health services showed a slight increase from 24 to 26 facilities, not statistically significant (*p* = 0.112). The use of guidelines to guide routine service provision remained consistent, with 24 of 26 facilities reporting their use during both periods no statistically significant difference, *p* = 0.859).

In terms of strategies to overcome service disruptions, the deployment of telemedicine increased only slightly from 20 to 21 facilities, which was not statistically significant (*p* = 0.999). Task shifting to community services declined from 33 to 27 facilities, but this was not statistically significant. Triaging to identify service priorities remained unchanged (26 facilities, *p* = 0.321). (For details see Supplementary Table A.1) (see Table 3).

**Table 3:** Health system functioning by phase of pandemic

**Variables**	**Pandemic period**		
	**During Pandemic** **(# of facilities that responded Yes)**	**Post-Pandemic** **(# of facilities that responded Yes)**	*p*-value *
Has your country defined a national SRH essential health services package (prior to the COVID-19 pandemic)? (*N* = 43)	42	37	0.165
Has your country identified a core set of essential health services to be maintained during the COVID-19 pandemic? (*N* = 48)	40	38	0.513
Is there additional government funding allocated to assuring essential health services? (*N* = 48)	24	26	0.112
Are the guidelines being used to guide the routine service provision? (*N* = 26)	24	24	0.859
***Approaches used to overcome disruptions***			
Telemedicine Deployment (N = 45)	20	21	0.999
Task Shifting to Community Services (*N* = 45)	33	27	NA
Triaging to Identify Priorities (*N* = 45)	26	26	0.321

*NA* Not applicable

^*^A multilevel longitudinal logistic model with "country" as a random effect was used to assess the significance of relevant effects, as indicated by *p*-values

Qualitative findings revealed that telemedicine and digital counseling were widely adopted in China and Brazil to mitigate service disruptions. In Kenya, however, limited infrastructure and internet access constrained the effectiveness of remote service delivery. Community engagement emerged as a key strategy in China and Brazil, where local networks helped disseminate SRH information and facilitate access to medicines. These adaptive responses were often initiated at the facility level, underscoring the importance of local innovation in crisis contexts.

### Family planning service provision

The analysis of Family Planning services data highlights slight improvements were seen in the presence of national family planning guidelines, checklists, job-aids, referrals, and separate rooms for family planning services; however, none of these changes were statistically significant (*p*-values > 0.05). (see Table 4). The availability of written information on contraceptive methods remained stable. Similarly, there were no statistically significant changes in the availability of contraceptive commodities, training in family planning and adolescent SRH, or the presence of both male and female service providers. Supplementary Table A.2 provides detailed information on Family Planning services. Overall, the data indicate that the variables of time, country, and their interaction did not significantly impact the outcomes. This underscores the necessity for ongoing efforts to improve family planning services and support (for details see Supplementary Table A.1).

**Table 4:** Disruptions to SRHR Services by pandemic timing: family planning, safe abortion, and GBV response

Pandemic period			
Facility level information*	**During pandemic (# of facilities that responded Yes)**	**Post-pandemic (# of facilities that responded Yes)**	***p*-value**
**Family planning services**			
Is the National guidelines present in the facility? (*N* = 42)	35	36	0.325
Job aids available in facility (ex. checklist, flow charts, algorithms, pictograms) (*N* = 41)	37	36	0.574
Any referrals for services to other healthcare facilities every month? (*N* = 42)	5	7	0.194
Written information and materials available, so that users can read or take materials home to read (*N* = 43)	21	23	0.272
Does this facility continue to stock commodities at this site? (*N* = 43)	42	41	0.663
Have the service providers received any training in the last 6 months? (*N* = 43)	21	20	0.293
**Safe abortion services**			
Is the National guidelines present in the facility? (*N* = 41)	19	23	0.199
Job aids available in facility (ex. checklist, flow charts, algorithms, pictograms) (*N* = 41)	20	27	0.345
Any referrals for services to other healthcare facilities every month? (*N* = 41)	8	10	0.285
Written information and materials available, so that users can read or take materials home to read (*N* = 39)	13	11	0.146
Does this facility continue to stock commodities at this site? (*N* = 43)	31	32	0.309
Have the service providers received any training in the last 6 months? (*N* = 43)	13	15	0.771
**Gender-based violence**			
Is the National guidelines present in the facility? (*N* = 40)	22	23	0.932
Job aids available in facility (ex. checklist, flow charts, algorithms, pictograms) (*N* = 39)	18	23	0.329
Any referrals for services to other healthcare facilities every month? (*N* = 39)	26	26	0.633
Does this facility continue to stock tetanus toxoid at this site? (*N* = 39)**	26	29	< 0.01
Have the service providers received training on responding to sexual violence in the last 6 months? (*N* = 39)	13	12	0.692

^*^A multilevel longitudinal logistic model with "country" as a random effect was used to assess the significance of relevant effects, as indicated by *p*-values

^**^Only information about emergency contraception is provided here, details of other commodities is in Supplementary table A1

Qualitative data from Brazil and China revealed that contraception was deprioritized during the pandemic, with women reporting difficulty accessing contraceptive methods and information. In Brazil, some women resorted to switching methods without medical guidance due to limited availability. In China, participants expressed support for expanding telehealth to improve contraceptive access, especially during emergencies.

### Abortion services

The analysis of Abortion services data shows slight improvements over time in the presence of national abortion guidelines, abortion care checklists, job-aids, referrals, and separate rooms for abortion services (See Table 4). The availability of written information on abortion methods and stock of abortion commodities remained stable. The availability of osmotic dilators and the gender diversity of service providers improved. Other areas such as training in abortion services and the availability of out-of-stock medicines remained at the same levels and showed no statistically significant changes (for details see Supplementary Table A.3).

In China and Kenya, qualitative interviews revealed that abortion services were among the most disrupted due to staff redeployment and facility closures. Women reported visiting multiple facilities before finding one that offered abortion care. Providers noted that abortion services were suspended in some locations, forcing clients to seek care elsewhere.

### Gender-based violence services

The analysis of GBV data shows the presence of national guidelines for domestic and sexual violence improved slightly, but not significantly. The availability of checklists and job-aids for domestic violence significantly increased (*p* < 0.05). (See Table 4). The provision of care and management for domestic violence, including medical care, psychological support, and referrals, showed some improvements, but these were not statistically significant.

The availability of post-rape care services (e.g., emergency contraception, HIV PEP, and STI prophylaxis) remained stable with no statistically significant changes. Training for healthcare providers on responding to intimate partner and sexual violence showed statistically significant improvements (*p* < 0.05). Supplementary Table A.4 provides more information (see Table 4).

Qualitative data from Kenya highlighted that stigma and fear of community backlash discouraged some women from seeking GBV-related services. Providers reported emotional strain in managing GBV cases during the pandemic, especially in the absence of psychosocial support systems.

### STI/HIV services

The analysis of STI-HIV data highlights that slight improvements were observed in the availability of HIV counseling/testing services, national HIV counseling/testing guidelines, and HIV rapid test kits, but none of these changes were statistically significant. Similarly, the availability of condoms, syphilis rapid testing, and HIV rapid testing showed minor improvements, but were not statistically significant. The availability of urine rapid tests for pregnancy showed a statistically significant improvement (*p* < 0.05). Training in STI diagnosis and treatment, privacy in HIV/STI testing and counseling, and availability of national guidelines remained stable. Overall, time, country, and their interaction did not significantly impact outcomes. Supplementary Table A.5 provides additional details (see Table 5).

**Table 5:** Disruptions to HIV and STI services by pandemic phase

**Variables**	**Pandemic period**		
	**During pandemic** **(# of facilities that responded Yes)**	**Post-pandemic** **(# of facilities that responded Yes)**	***p*-value**
Does this facility offer HIV counselling and testing services (*N* = 39)	35	34	0.501
Do you have the national HIV counselling and testing guidelines available in this facility today? (*N* = 39)	31	34	0.474
Does this facility have HIV rapid test kits (with valid expiration date) in stock today, ready for client testing? (*N* = 39)	30	33	0.968
Does this facility offer any of the following tests on-site? Syphillis Rapid Testing (*N* = 35)	27	29	0.583
HIV rapid testing (*N* = 36)	33	32	0.995
Dry Blood Spot (DBS) collection for HIV viral load or EID (*N* = 36)	22	22	0.489
Do providers in this facility diagnose STIs? (*N* = 39)	39	39	NA
Do you have the national guidelines for the diagnosis and treatment of STIs available in this facility today? (*N* = 39)	33	34	0.325
Have you or any provider(s) of STI services received any training in STI diagnosis and treatment in the last two years? (*N* = 39)	19	27	0.535
Do you have guidelines for partner notification and contact tracing available? Partner Notification (PN) (for HIV) yes, national guidelines (*N* = 38)	29	33	0.369

Effect of time (endline vs. baseline) in key variables of Family Planning form

A multilevel longitudinal logistic model, with "country" as a random effect, was used to assess significance of relevant effects (*p* values)

*NA* Not applicable

In Kenya, qualitative interviews revealed that STI services were disrupted due to staff shortages and fear of contagion. Providers reported difficulty maintaining routine STI screening and treatment, and some clients avoided facilities altogether. In China, women expressed concern about accessing STI medications during lockdowns, while in Brazil, limited provider availability hindered routine STI care.

## Discussion

The COVID-19 pandemic has had substantive impacts on global health systems, particularly in the provision of SRH services. The findings from the WHO/HRP's multi-country study highlight both the profound vulnerabilities and remarkable resilience of health systems during the COVID-19 pandemic, with service disruptions and mitigation strategies varying significantly by country context and resource level. Importantly, the pandemic did not create these weaknesses but rather exposed and magnified pre-existing systemic challenges, such as chronic underfunding, fragile supply chains, insufficient workforce support, and inequities in service access that have long constrained SRH service delivery. This demonstrates that health systems were already vulnerable, and the crisis acted as a stress test revealing their structural fragility. The findings suggest that while foundational policies and service delivery frameworks were in place before and during the pandemic, there was limited evolution or scaling up of these efforts in the post-pandemic period. The minimal changes in funding, telemedicine use, and triaging practices point to a potential gap between policy intent and implementation. This gap underscores that preparedness plans alone are insufficient without mechanisms for rapid operationalization and accountability One potential reason for few statistically significant results could be because the post-pandemic period was ultimately too close to the pandemic period and it took countries more time to recover than the study timeline (9–12 months, country-dependent). Moreover, the decline in task shifting may reflect a re-centralization of services or resource constraints. Overall, these findings reinforce the urgent need to institutionalize adaptive, flexible, and equity-focused strategies that can sustain SRH services during any future outbreak, not just COVID-19.

Across China [18], Brazil [19], and Kenya [20], several key mechanisms helped mitigate pandemic-related challenges to maintain SRH services and reach patients. Telemedicine emerged as a central strategy in China and Brazil, enabling remote consultations, triaging, and continuity of care, especially for antenatal and contraceptive services. China leveraged digital platforms like WeChat, while Brazil used tele-orientation and call centers in some municipalities. Kenya, in contrast, relied more on in-person service delivery, with limited telehealth infrastructure. Task-sharing and role adaptation were widely used: China redeployed staff to fever clinics, Brazil reassigned general practitioners and nurses to SRH tasks, and Kenya leaned heavily on nurses and midwives to sustain services amid workforce shortages. Triaging systems were implemented to prioritize urgent cases and redirect patients, particularly in China and Brazil. Community engagement also played a vital role, especially in China, where local health workers bridged communication gaps and supported service delivery.

However, differences in implementation and effectiveness were evident. China’s centralized and technologically integrated response enabled rapid deployment of telehealth and community-based outreach. Brazil’s response varied by city, with some municipalities effectively using digital tools and others struggling due to infrastructure limitations and political discord. Kenya faced significant psychosocial challenges among healthcare workers, with limited institutional support and informal coping mechanisms such as avoidance of testing and reliance on faith. Unlike China and Brazil, Kenya’s mitigation efforts focused more on maintaining physical service delivery under stress, highlighting the need for future responses to integrate mental health support and clear communication strategies alongside service continuity. These cross-country differences underscore the importance of tailoring interventions to local contexts and resource levels.

Three critical intervention areas emerge from our findings: workforce support strategies to address staffing crises, particularly in high-burden areas; supply chain reinforcement for essential commodities in low-resource settings; and adaptive service delivery models to overcome access barriers. The stark contrast between high-income countries' technology-driven responses and low-resource settings' community-based adaptations underscores the need for context-specific solutions. Future preparedness strategies should therefore operate at two levels: first, ensuring continuity of essential SRH services during outbreak response (crisis management), and second, strengthening long-term health system capacity to withstand similar shocks. Future pandemic preparedness efforts must address these systemic vulnerabilities while building on successful adaptations, with particular attention to maintaining essential reproductive health services and strengthening emergency response capabilities. These findings highlight both the fragility of global health systems and the remarkable adaptability demonstrated under crisis conditions.

The initial waves of COVID-19 saw health systems overwhelmed, with resources diverted to manage the pandemic, leading to reduced availability of essential SRH services [2, 11]. This diversion not only disrupted SRH access during COVID-19 but also demonstrated how quickly critical but often under-prioritized services can become marginalized when competing demands arise. This was consistent with previous outbreaks like Zika and Ebola, where similar disruptions and delayed recovery were observed [5, 6]. Taken together, these parallels suggest that SRH services remain one of the most vulnerable domains of health care in emergencies, highlighting a recurring systemic weakness that transcends individual outbreaks. The anticipated increase in unintended pregnancies due to lockdown-related disruptions underscores the need for resilient SRH service delivery systems [11]. The results also indicate a decrease in the number of pregnant women attending clinics, which may be attributed to concerns about potential negative side effects and pregnancy outcomes, similar to the fears experienced during the Zika and Ebola outbreaks. The availability of contraceptive commodities and abortion methods remained stable, indicating that while health systems attempted to maintain these services, the challenges posed by the pandemic were substantial. The proportion of patients whose most important concern was access to their preferred method of contraception increased significantly over time. While the availability of family planning services did not change significantly, and perception of access and actual access to preferred contraceptive methods became more challenging post-pandemic.

The provision of STI and HIV services showed minor improvements, particularly in the availability of rapid test kits and condoms. However, these changes were not significant enough to indicate a robust recovery. GBV services saw some improvements, particularly in the availability of checklists and job-aids for domestic violence. Training for healthcare providers on responding to intimate partner and sexual violence showed significant progress, indicating a positive shift towards capacity building. However, the provision of post-rape care services remained stable, suggesting that while there were efforts to enhance GBV support, more work is needed to ensure comprehensive care. While there was a significant decrease in visits related to violence, this doesn’t necessarily indicate a meaningful difference in the prevalence of cases of violence experienced by clients seeking violence-related visits during and at endline. This demonstrates the need for continuing and expanding access to GBV services, especially during health crises such as an epidemic or pandemic. The persistence of these gaps further illustrates how systemic weaknesses in SRH service integration with broader health and social protection systems amplify vulnerabilities during emergencies.

The study's multi-country approach provided insights into regional variations in SRH service likely disruptions and degree of recovery. These variations underscore the importance of context-specific strategies in health emergency responses. The post-pandemic recovery of SRH services varied across regions and facilities. While certain areas, such as STI testing (particularly pregnancy rapid tests) and GBV provider training and tools, showed improvements in service availability and quality, other services continued to face significant barriers. Resilience strategies including telemedicine, task-sharing, and community engagement, helped mitigate disruptions, though their implementation remained uneven across countries. The study's findings highlight the need for targeted interventions to address these disparities and ensure equitable access to SRH services. These disparities also signal that global health preparedness must move beyond a ‘one-size-fits-all’ approach and instead incorporate flexible frameworks that can be adapted to country- and facility-level realities.

This study has limitations. First, the repeated cross-sectional design limits the ability to infer causality or track individual-level changes over time. Second, due to the unpredictable and varied timing of COVID-19 waves across countries, only seven of the nine participating countries could define clear cut-off points for “during” and “post-pandemic” periods; consequently, data from Ghana and Burkina Faso were excluded from the final analysis. Third, while efforts were made to select diverse health facilities, the results may not be fully generalizable to all regions within participating countries. Fourth, the use of *p* < 0.10 as a threshold for significance is a more liberal criterion and not typically considered statistically significant in conventional terms, although employed in its exploratory context provided some useful preliminary insights. Lastly, social desirability bias may have affected self-reported data from clients and providers, particularly regarding sensitive topics such as gender-based violence and abortion services.

## Conclusion

This research provides valuable insights into the impact of COVID-19 on SRH services and the subsequent recovery efforts. While there were some improvements in service availability and quality, the effects of the pandemic's were significant, causing recovery across the services to remain slow and pervasive. The findings underscore the need for resilient health systems that can maintain essential services during crises. Targeted interventions, capacity building, and sustained government support are essential in ensuring equitable access to SRH services. Importantly, the pandemic highlighted long-standing systemic challenges such as fragile supply chains, chronic underfunding, inadequate workforce protections, and inequities in service access that extend beyond COVID-19 and continue to undermine SRH care globally.

This study also revealed that countries employed a range of mitigation mechanisms such as telemedicine, task-sharing, triaging, and community engagement to maintain SRH services during the pandemic, with varying degrees of success. China and Brazil leveraged digital platforms and remote consultations, while Kenya relied more on in-person service delivery and informal coping strategies among healthcare workers. These differences underscore the importance of tailoring interventions to local contexts and resource levels. The implications of these findings are twofold: first, in the immediate context of COVID-19, they stress the importance of institutionalizing flexible, adaptive measures (e.g., telemedicine, task shifting, supply chain reinforcement) to protect SRH during emergencies; second, at the broader health system level, they reveal the urgency of embedding SRH services within national preparedness and response frameworks so they are not sidelined during future crises.

As with previous outbreaks such as Zika and Ebola, COVID-19 demonstrated that SRH services are often among the first to be disrupted, underscoring their systemic vulnerability in times of health system stress. Ensuring continuity of SRH care must therefore be treated as an essential component of epidemic and pandemic response planning.

As new challenges like post-COVID-19 complications on menstrual abnormalities and infertility arise [21] and the world continues to navigate the post-pandemic landscape, these lessons go beyond COVID-19, offering critical guidance for safeguarding SRH during any future outbreak. Ultimately, strengthening the resilience of health systems through sustained investment, political commitment, and equity-centered approaches will be key to protecting the sexual and reproductive health and rights of women and girls globally.

## Supplementary Information

Supplementary Material 1.

## Footnotes

1 Sexual and reproductive health (SRH) services constitute a comprehensive spectrum of care that extends beyond basic medical interventions to encompass accurate information, counselling, and evidence-based sexuality education. These services address diverse needs, including the promotion of sexual well-being and satisfaction, as well as the prevention, detection, and management of sexual and gender-based violence. Central components include the provision of a range of safe and effective contraceptive methods, high-quality antenatal, intrapartum, and postnatal care, and access to abortion services. Furthermore, SRH services play a critical role in the prevention, diagnosis, and treatment of infertility, sexually transmitted and reproductive tract infections including HIV and reproductive cancers. Link: bit.ly/3IGFHHb.

2 A total of 4,346 clients were interviewed across all participating countries during the study period. However, due to the unpredictable timing and recurrence of COVID-19 waves, it was not possible to establish cut-off points for the 'during' and 'post-pandemic' periods in Ghana and Burkina Faso. Consequently, these two countries were excluded from the final analysis, which included data from 2,292 clients.
