# Supplementary material for: Evidence on sexual and reproductive health service delivery during and post COVID-19: a multi-country facility assessment
Source: Reprod Health. 2025 Nov 20;22(Suppl 3):233. doi: 10.1186/s12978-025-02185-w (PMC12632035; doi:10.1186/s12978-025-02185-w)
Supplement: Supplementary file 1 — Supplementary Material 1. [file 12978_2025_2185_MOESM1_ESM.docx]

**Supplementary Tables**

***Supplementary table A1: Causes of disruption of SRH services and mitigation strategies***

| *Health centers in participating countries* | | |
| --- | --- | --- |
|  | ***N (responding Yes)*** | ***TOTAL***  ***(%)*** |
| *Disruption Causes* | | |
| *Closure of Outpatient Services (N=49)* | 16 | 32.7% |
| *Insufficient Staff (N=49)* | 18 | 36.7% |
| *Staff Deployed to COVID-19 (N=49)* | 24 | 49.0% |
| *Insufficient PPE (N=49)* | 15 | 30.6% |
| *Stock Out of Essential Medicines (N=49)* | 9 | 18.4% |
| *Public Transport Lockdowns (N=49)* | 29 | 60.4% |
| *Mitigation Strategies* | | |
| *Telemedicine Deployment (N=49)* | 23 | 46.9% |
| *Task Shifting (N=49)* | 36 | 73.5% |
| *Novel Supply Chain (N=49)* | 23 | 46.9% |
| *Triaging Priorities (N=49)* | 28 | 57.1% |
| *Redirection of Patients (N=49)* | 23 | 46.9% |
| *Community Outreach (N=49)* | 24 | 49.0% |
| *Gov. Removal of User Fees (N=47)* | 4 | 8.5% |

**Supplementary table A2: FAMILY PLANNING SERVICES**

| CROSSTABULATIONS (post pandemic] vs. during pandemic) | | | | | |
| --- | --- | --- | --- | --- | --- |
|  |  |  |  |  |  |
| Is the National family planning guidelines present in the facility? | **During pandemic vs. Post pandemic** | | | | |
|  |  |  |  |  |  |
|  |  | | Post | | Total |
|  |  |  | NO | YES |  |
|  | During | NO | 4 | 3 | 7 |
|  |  | YES | 2 | 33 | 35 |
|  | Total | | 6 | 36 | 42 |
| *p-value for time effect: 0.325* | | | | | |
| Are there any family planning check-lists and/or job-aids available in the facility? | **During pandemic vs. Post pandemic** | | | | |
|  |  |  |  |  |  |
|  |  | | Post | | Total |
|  |  |  | NO | YES |  |
|  | During | NO | 4 | 0 | 4 |
|  |  | YES | 1 | 36 | 37 |
|  | Total | | 5 | 36 | 41 |
| *p-value for time effect: 0.574* | | | | | |
| Any referrals for family planning services to other healthcare facilities per month? | **During pandemic vs. Post pandemic** | | | | |
|  |  |  |  |  |  |
|  |  | | Post | | Total |
|  |  |  | NO | YES |  |
|  | During | NO | 34 | 3 | 37 |
|  |  | YES | 1 | 4 | 5 |
|  | Total | | 35 | 7 | 42 |
| *p-value for time effect: 0.194* | | | | | |
| There is a separate room for FP/contraception services (e.g. for private examination, insertion/removal of IUDs, counselling room): | **During pandemic vs. Post pandemic** | | | | |
|  |  |  |  |  |  |
|  |  | | Post | | Total |
|  |  |  | NO | YES |  |
|  | During | NO | 4 | 5 | 9 |
|  |  | YES | 1 | 33 | 34 |
|  | Total | | 5 | 38 | 43 |
| *p-value for time effect: 0.244* | | | | | |
| There are separate waiting rooms, especially for adolescents: | **During pandemic vs. Post pandemic** | | | | |
|  |  |  |  |  |  |
|  |  | | Post | | Total |
|  |  |  | NO | YES |  |
|  | During | NO | 31 | 4 | 35 |
|  |  | YES | 4 | 4 | 8 |
|  | Total | | 35 | 8 | 43 |
| *p-value for time effect: 0.645* | | | | | |
| There are written information and materials available on the various contraceptive methods, so that users can take materials home to read: | **During pandemic vs. Post pandemic** | | | | |
|  |  |  |  |  |  |
|  |  | | Post | | Total |
|  |  |  | NO | YES |  |
|  | During | NO | 16 | 6 | 22 |
|  |  | YES | 4 | 17 | 21 |
|  | Total | | 20 | 23 | 43 |
| *p-value for time effect: 0.272* | | | | | |
| There are posters about violence against women (e.g. that it is not acceptable, can cause harm/health problems, you can discuss with your provider) and/or leaflets available: | **During pandemic vs. Post pandemic** | | | | |
|  |  |  |  |  |  |
|  |  | | Post | | Total |
|  |  |  | NO | YES |  |
|  | During | NO | 24 | 10 | 34 |
|  |  | YES | 2 | 7 | 9 |
|  | Total | | 26 | 17 | 43 |
| *p-value for time effect: 0.808* | | | | | |
| Does this facility continue to stock contraceptive commodities at this site? | **During pandemic vs. Post pandemic** | | | | |
|  |  |  |  |  |  |
|  |  | | Post | | Total |
|  |  |  | NO | YES |  |
|  | During | NO | 1 | 0 | 1 |
|  |  | YES | 1 | 41 | 42 |
|  | Total | | 2 | 41 | 43 |
| *p-value for time effect: 0.663* | | | | | |
| Have the family planning service providers received any training in family planning in the last 6 months? | **During pandemic vs. Post pandemic** | | | | |
|  |  |  |  |  |  |
|  |  | | Post | | Total |
|  |  |  | NO | YES |  |
|  | During | NO | 16 | 6 | 22 |
|  |  | YES | 7 | 14 | 21 |
|  | Total | | 23 | 20 | 43 |
| *p-value for time effect: 0.293* | | | | | |
| Have the family planning service providers received any training in adolescent sexual and reproductive health (including family planning) in the last 6 months? | **During pandemic vs. Post pandemic** | | | | |
|  |  |  |  |  |  |
|  |  | | Post | | Total |
|  |  |  | NO | YES |  |
|  | During | NO | 25 | 5 | 30 |
|  |  | YES | 5 | 7 | 12 |
|  | Total | | 30 | 12 | 42 |
| *p-value for time effect: 0.475* | | | | | |
| Sex of Service providers available? | **During pandemic vs. Post pandemic** | | | | |
|  |  |  |  |  |  |
|  |  | | Post | | Total |
|  |  |  | NO | YES |  |
|  | During | NO | 15 | 4 | 19 |
|  |  | YES | 9 | 14 | 23 |
|  | Total | | 24 | 18 | 42 |
| *p-value for time effect: 0.167* | | | | | |

*Effect of time (endline vs. baseline) in key variables of Family Planning form.*

*A multilevel longitudinal logistic model, with "country" as a random effect, was used to assess significance of relevant effects (p values).*

**Supplementary table A3: ABORTION CARE SERVICES**

| CROSSTABULATIONS (post pandemic] vs. during pandemic) | | | | | |
| --- | --- | --- | --- | --- | --- |
|  |  |  |  |  |  |
| Is the National abortion guidelines present in the facility? | **During pandemic vs. Post pandemic** | | | | |
|  |  |  |  |  |  |
|  |  | | Post | | Total |
|  |  |  | NO | YES |  |
|  | During | NO | 16 | 6 | 22 |
|  |  | YES | 2 | 17 | 19 |
|  | Total | | 18 | 23 | 41 |
| p-value for time effect: 0.199 |  |  |  |  |  |
|  |  |  |  |  |  |
| Are there any safe abortion check-lists and/or job-aids available in the facility? | **During pandemic vs. Post pandemic** | | | | |
|  |  |  |  |  |  |
|  |  | | Post | | Total |
|  |  |  | NO | YES |  |
|  | During | NO | 13 | 8 | 21 |
|  |  | YES | 1 | 19 | 20 |
|  | Total | | 14 | 27 | 41 |
| p-value for time effect: 0.345 |  |  |  |  |  |
|  |  |  |  |  |  |
| Any referrals for abortion services to other healthcare facilities per month? | **During pandemic vs. Post pandemic** | | | | |
|  |  |  |  |  |  |
|  |  | | Post | | Total |
|  |  |  | NO | YES |  |
|  | During | NO | 29 | 4 | 33 |
|  |  | YES | 2 | 6 | 8 |
|  | Total | | 31 | 10 | 41 |
| p-value for time effect: 0.285 |  |  |  |  |  |
|  |  |  |  |  |  |
| What is the major reason of the referrals? Lack or absence of specific procedures to treat women seeking postabortion services | **During pandemic vs. Post pandemic** | | | | |
|  |  |  |  |  |  |
|  |  | | Post | | Total |
|  |  |  | NO | YES |  |
|  | During | NO | 3 | 0 | 3 |
|  |  | YES | 0 | 1 | 1 |
|  | Total | | 3 | 1 | 4 |
| p-value for time effect: 0.653 |  |  |  |  |  |
|  |  |  |  |  |  |
| What is the major reason of the referrals? Lack or absence of Medical Termination (pharmacological and/or surgical) of the pregnancy | **During pandemic vs. Post pandemic** | | | | |
|  |  |  |  |  |  |
|  |  | | Post | | Total |
|  |  |  | NO | YES |  |
|  | During | NO | 1 | 0 | 1 |
|  |  | YES | 0 | 3 | 3 |
|  | Total | | 1 | 3 | 4 |
| p-value for time effect: 0.404 |  |  |  |  |  |
|  |  |  |  |  |  |
| What is the major reason of the referrals? Other reasons | **During pandemic vs. Post pandemic** | | | | |
|  |  |  |  |  |  |
|  |  | | Post | | Total |
|  |  |  | NO | YES |  |
|  | During | NO | 2 | 0 | 2 |
|  |  | YES | 1 | 1 | 2 |
|  | Total | | 3 | 1 | 4 |
| p-value for time effect: 0.986 |  |  |  |  |  |
|  |  |  |  |  |  |
| For adult clients: There is a separate room for abortion services for clients (e.g. for private examination): | **During pandemic vs. Post pandemic** | | | | |
|  |  |  |  |  |  |
|  |  | | Post | | Total |
|  |  |  | NO | YES |  |
|  | During | NO | 15 | 8 | 23 |
|  |  | YES | 2 | 15 | 17 |
|  | Total | | 17 | 23 | 40 |
| p-value for time effect: 0.416 |  |  |  |  |  |
|  |  |  |  |  |  |
| a) For Adolescents: There are separate waiting rooms: | **During pandemic vs. Post pandemic** | | | | |
|  |  |  |  |  |  |
|  |  | | Post | | Total |
|  |  |  | NO | YES |  |
|  | During | NO | 36 | 1 | 37 |
|  |  | YES | 1 | 2 | 3 |
|  | Total | | 37 | 3 | 40 |
| p-value for time effect: 0.691 |  |  |  |  |  |
|  |  |  |  |  |  |
| There are written information and materials available on the various safe abortion methods, so that users can take materials home to read: | **During pandemic vs. Post pandemic** | | | | |
|  |  |  |  |  |  |
|  |  | | Post | | Total |
|  |  |  | NO | YES |  |
|  | During | NO | 24 | 2 | 26 |
|  |  | YES | 4 | 9 | 13 |
|  | Total | | 28 | 11 | 39 |
| p-value for time effect: 0.146 |  |  |  |  |  |
|  |  |  |  |  |  |
| Does the facility continue to stock abortion commodities? | **During pandemic vs. Post pandemic** | | | | |
|  |  |  |  |  |  |
|  |  | | Post | | Total |
|  |  |  | NO | YES |  |
|  | During | NO | 6 | 5 | 11 |
|  |  | YES | 4 | 27 | 31 |
|  | Total | | 10 | 32 | 42 |
| p-value for time effect: 0.309 |  |  |  |  |  |
|  |  |  |  |  |  |
| Any of the following medicines were out of stock in this facility during the last 6 months? Misoprostol: | **During pandemic vs. Post pandemic** | | | | |
|  |  |  |  |  |  |
|  |  | | Post | | Total |
|  |  |  | NO | YES |  |
|  | During | NO | 20 | 9 | 29 |
|  |  | YES | 8 | 5 | 13 |
|  | Total | | 28 | 14 | 42 |
| p-value for time effect: 0.255 |  |  |  |  |  |
|  |  |  |  |  |  |
| Any of the following medicines were out of stock in this facility during the last 6 months? Osmotic dilators: | **During pandemic vs. Post pandemic** | | | | |
|  |  |  |  |  |  |
|  |  | | Post | | Total |
|  |  |  | NO | YES |  |
|  | During | NO | 25 | 6 | 31 |
|  |  | YES | 4 | 7 | 11 |
|  | Total | | 29 | 13 | 42 |
| p-value for time effect: <0.001 |  |  |  |  |  |
|  |  |  |  |  |  |
| Any of the following medicines were out of stock in this facility during the last 6 months? Mifepristone: | **During pandemic vs. Post pandemic** | | | | |
|  |  |  |  |  |  |
|  |  | | Post | | Total |
|  |  |  | NO | YES |  |
|  | During | NO | 21 | 8 | 29 |
|  |  | YES | 6 | 6 | 12 |
|  | Total | | 27 | 14 | 41 |
| p-value for time effect: 0.838 |  |  |  |  |  |
|  |  |  |  |  |  |
| Any of the following medicines were out of stock in this facility during the last 6 months? Analgesics: | **During pandemic vs. Post pandemic** | | | | |
|  |  |  |  |  |  |
|  |  | | Post | | Total |
|  |  |  | NO | YES |  |
|  | During | NO | 27 | 5 | 32 |
|  |  | YES | 7 | 3 | 10 |
|  | Total | | 34 | 8 | 42 |
| p-value for time effect: 0.487 |  |  |  |  |  |
|  |  |  |  |  |  |
| Any of the following medicines were out of stock in this facility during the last 6 months? Anxiolytics: | **During pandemic vs. Post pandemic** | | | | |
|  |  |  |  |  |  |
|  |  | | Post | | Total |
|  |  |  | NO | YES |  |
|  | During | NO | 29 | 5 | 34 |
|  |  | YES | 5 | 2 | 7 |
|  | Total | | 34 | 7 | 41 |
| p-value for time effect: 0.902 |  |  |  |  |  |
|  |  |  |  |  |  |
| Any of the following medicines were out of stock in this facility during the last 6 months? Antibiotics: | **During pandemic vs. Post pandemic** | | | | |
|  |  |  |  |  |  |
|  |  | | Post | | Total |
|  |  |  | NO | YES |  |
|  | During | NO | 27 | 4 | 31 |
|  |  | YES | 6 | 5 | 11 |
|  | Total | | 33 | 9 | 42 |
| p-value for time effect: 0.358 |  |  |  |  |  |
|  |  |  |  |  |  |
| Any of the following medicines were out of stock in this facility during the last 6 months? Fluids (saline, sodium lactate, glucose): | **During pandemic vs. Post pandemic** | | | | |
|  |  |  |  |  |  |
|  |  | | Post | | Total |
|  |  |  | NO | YES |  |
|  | During | NO | 29 | 5 | 34 |
|  |  | YES | 5 | 3 | 8 |
|  | Total | | 34 | 8 | 42 |
| p-value for time effect: 0.558 |  |  |  |  |  |
|  |  |  |  |  |  |
| Any of the following medicines were out of stock in this facility during the last 6 months? Lidocaine for paracervical block: | **During pandemic vs. Post pandemic** | | | | |
|  |  |  |  |  |  |
|  |  | | Post | | Total |
|  |  |  | NO | YES |  |
|  | During | NO | 27 | 5 | 32 |
|  |  | YES | 4 | 6 | 10 |
|  | Total | | 31 | 11 | 42 |
| p-value for time effect: 0.991 |  |  |  |  |  |
|  |  |  |  |  |  |
| Appropriate antagonists to medications used for pain: | **During pandemic vs. Post pandemic** | | | | |
|  |  |  |  |  |  |
|  |  | | Post | | Total |
|  |  |  | NO | YES |  |
|  | During | NO | 28 | 5 | 33 |
|  |  | YES | 4 | 5 | 9 |
|  | Total | | 32 | 10 | 42 |
| p-value for time effect: 0.542 |  |  |  |  |  |
|  |  |  |  |  |  |
| Uterotonics (oxytocin, misoprostol or ergometrine) | **During pandemic vs. Post pandemic** | | | | |
|  |  |  |  |  |  |
|  |  | | Post | | Total |
|  |  |  | NO | YES |  |
|  | During | NO | 28 | 4 | 32 |
|  |  | YES | 5 | 5 | 10 |
|  | Total | | 33 | 9 | 42 |
|  |  |  |  |  |  |
| Antiseptic solution (non-alcohol based) to prepare the cervix | **During pandemic vs. Post pandemic** | | | | |
|  |  |  |  |  |  |
|  |  | | Post | | Total |
|  |  |  | NO | YES |  |
|  | During | NO | 27 | 5 | 32 |
|  |  | YES | 7 | 3 | 10 |
|  | Total | | 34 | 8 | 42 |
| p-value for time effect: 0.381 |  |  |  |  |  |
|  |  |  |  |  |  |
| Sterilization or high-level disinfection solutions and materials: | **During pandemic vs. Post pandemic** | | | | |
|  |  |  |  |  |  |
|  |  | | Post | | Total |
|  |  |  | NO | YES |  |
|  | During | NO | 28 | 4 | 32 |
|  |  | YES | 6 | 4 | 10 |
|  | Total | | 34 | 8 | 42 |
| p-value for time effect: 0.401 |  |  |  |  |  |
|  |  |  |  |  |  |
| Have the abortion service providers received any training in safe abortion services in the last 6 months? | **During pandemic vs. Post pandemic** | | | | |
|  |  |  |  |  |  |
|  |  | | Post | | Total |
|  |  |  | NO | YES |  |
|  | During | NO | 22 | 5 | 27 |
|  |  | YES | 3 | 10 | 13 |
|  | Total | | 25 | 15 | 40 |
| p-value for time effect: 0.771 |  |  |  |  |  |
|  |  |  |  |  |  |
| Gender of service providers available (female) | **During pandemic vs. Post pandemic** | | | | |
|  |  |  |  |  |  |
|  |  | | Post | | Total |
|  |  |  | NO | YES |  |
|  | During | NO | 7 | 8 | 15 |
|  |  | YES | 4 | 15 | 19 |
|  | Total | | 11 | 23 | 34 |
| p-value for time effect: <0.001 | |  |  |  |  |
|  |  |  |  |  |  |
| Type of personnel (directly involved in safe abortion care) available at the health facility: OB/Gyn specialist: | **During pandemic vs. Post pandemic** | | | | |
|  |  |  |  |  |  |
|  |  | | Post | | Total |
|  |  |  | NO | YES |  |
|  | During | NO | 11 | 4 | 15 |
|  |  | YES | 3 | 20 | 23 |
|  | Total | | 14 | 24 | 38 |
| p-value for time effect: 0.945 |  |  |  |  |  |
|  |  |  |  |  |  |
| Type of personnel (directly involved in safe abortion care) available at the health facility: Medical doctor/ general practitioner: | **During pandemic vs. Post pandemic** | | | | |
|  |  |  |  |  |  |
|  |  | | Post | | Total |
|  |  |  | NO | YES |  |
|  | During | NO | 13 | 1 | 14 |
|  |  | YES | 6 | 16 | 22 |
|  | Total | | 19 | 17 | 36 |
| p-value for time effect: 0.752 |  |  |  |  |  |
|  |  |  |  |  |  |
| Type of personnel (directly involved in safe abortion care) available at the health facility: Nurse: | **During pandemic vs. Post pandemic** | | | | |
|  |  |  |  |  |  |
|  |  | | Post | | Total |
|  |  |  | NO | YES |  |
|  | During | NO | 12 | 3 | 15 |
|  |  | YES | 7 | 14 | 21 |
|  | Total | | 19 | 17 | 36 |
| p-value for time effect: 0.297 |  |  |  |  |  |
|  |  |  |  |  |  |
| Type of personnel (directly involved in safe abortion care) available at the health facility: Midwife: | **During pandemic vs. Post pandemic** | | | | |
|  |  |  |  |  |  |
|  |  | | Post | | Total |
|  |  |  | NO | YES |  |
|  | During | NO | 10 | 3 | 13 |
|  |  | YES | 6 | 17 | 23 |
|  | Total | | 16 | 20 | 36 |
| p-value for time effect: 0.558 |  |  |  |  |  |
|  |  |  |  |  |  |
| Type of personnel (directly involved in safe abortion care) available at the health facility: Other health workers ( | **During pandemic vs. Post pandemic** | | | | |
|  |  |  |  |  |  |
|  |  | | Post | | Total |
|  |  |  | NO | YES |  |
|  | During | NO | 11 | 7 | 18 |
|  |  | YES | 2 | 13 | 15 |
|  | Total | | 13 | 20 | 33 |
| p-value for time effect: 0.583 |  |  |  |  |  |
|  |  |  |  |  |  |

*Effect of time (endline vs. baseline) in key variables of Family Planning form.*

*A multilevel longitudinal logistic model, with "country" as a random effect, was used to assess significance of relevant effects (p values).*

**Supplementary table A4: GENDER BASED VIOLENCE SERVICES**

| CROSSTABULATIONS (post pandemic] vs. during pandemic) | | | | | | |
| --- | --- | --- | --- | --- | --- | --- |
|  |  |  |  |  |  |  |
| Are the National guidelines for provision of health care to women subjected to domestic and/or sexual violence available in the facility? a) Domestic violence: | | **During pandemic vs. Post pandemic** | | | | |
|  |  |  |  |  |  |  |
|  |  |  | | Post | | Total |
|  |  |  |  | NO | YES |  |
|  |  | During | NO | 13 | 5 | 18 |
|  |  |  | YES | 4 | 18 | 22 |
|  |  | Total | | 17 | 23 | 40 |
| p-value for time effect: 0.932 | |  |  |  |  |  |
| Are the National guidelines for provision of health care to women subjected to domestic and/or sexual violence available in the facility? b) Sexual violence: | | **During pandemic vs. Post pandemic** | | | | |
|  |  |  |  |  |  |  |
|  |  |  | | Post | | Total |
|  |  |  |  | NO | YES |  |
|  |  | During | NO | 10 | 4 | 14 |
|  |  |  | YES | 0 | 24 | 24 |
|  |  | Total | | 10 | 28 | 38 |
| p-value for time effect: 0.458 | |  |  |  |  |  |
|  | |  |  |  |  |  |
| Are there any domestic and/or sexual violence check-lists and/or job-aids available in the facility?  a) Domestic violence: | | **During pandemic vs. Post pandemic** | | | | |
|  |  |  |  |  |  |  |
|  |  |  | | Post | | Total |
|  |  |  |  | NO | YES |  |
|  |  | During | NO | 16 | 7 | 23 |
|  |  |  | YES | 3 | 14 | 17 |
|  |  | Total | | 19 | 21 | 40 |
| p-value for time effect: <0.001 | |  |  |  |  |  |
| Are there any domestic and/or sexual violence check-lists and/or job-aids available in the facility?  b) Sexual violence: | | **During pandemic vs. Post pandemic** | | | | |
|  |  |  |  |  |  |  |
|  |  |  | | Post | | Total |
|  |  |  |  | NO | YES |  |
|  |  | During | NO | 15 | 6 | 21 |
|  |  |  | YES | 0 | 16 | 16 |
|  |  | Total | | 15 | 22 | 37 |
| p-value for time effect: 0.900 | |  |  |  |  |  |
|  |  |  |  |  |  |  |
|  |  |  |  |  |  |  |
| Does the facility provide the following aspects of care/management of domestic violence?  a) Ask/identify cases of domestic violence: | | **During pandemic vs. Post pandemic** | | | | |
|  |  |  |  |  |  |  |
|  |  |  | | Post | | Total |
|  |  |  |  | NO | YES |  |
|  |  | During | NO | 8 | 5 | 13 |
|  |  |  | YES | 0 | 25 | 25 |
|  |  | Total | | 8 | 30 | 38 |
| p-value for time effect: 0.522 | |  |  |  |  |  |
| Does the facility provide the following aspects of care/management of domestic violence?  b) Offer injury or other medical care to those who need it: | | **During pandemic vs. Post pandemic** | | | | |
|  |  |  |  |  |  |  |
|  |  |  | | Post | | Total |
|  |  |  |  | NO | YES |  |
|  |  | During | NO | 4 | 2 | 6 |
|  |  |  | YES | 1 | 32 | 33 |
|  |  | Total | | 5 | 34 | 39 |
| p-value for time effect: 0.852 | |  |  |  |  |  |
| Does the facility provide the following aspects of care/management of domestic violence?  c) Offer psychological support/crisis counselling/first-line support to those who disclose (internal referral) | | **During pandemic vs. Post pandemic** | | | | |
|  |  |  |  |  |  |  |
|  |  |  | | Post | | Total |
|  |  |  |  | NO | YES |  |
|  |  | During | NO | 9 | 3 | 12 |
|  |  |  | YES | 3 | 24 | 27 |
|  |  | Total | | 12 | 27 | 39 |
| p-value for time effect: 0.830 | |  |  |  |  |  |
| Does the facility provide the following aspects of care/management of domestic violence?  d) Refer to NGOs, counselling, or any other services outside the health facility that the woman might need | | **During pandemic vs. Post pandemic** | | | | |
|  |  |  |  |  |  |  |
|  |  |  | | Post | | Total |
|  |  |  |  | NO | YES |  |
|  |  | During | NO | 11 | 3 | 14 |
|  |  |  | YES | 0 | 25 | 25 |
|  |  | Total | | 11 | 28 | 39 |
| p-value for time effect: 0.801 | |  |  |  |  |  |
| Does the facility provide the following elements of post-rape care/care to survivors of sexual violence?  a) Injury treatment | | **During pandemic vs. Post pandemic** | | | | |
|  |  |  |  |  |  |  |
|  |  |  | | Post | | Total |
|  |  |  |  | NO | YES |  |
|  |  | During | NO | 5 | 0 | 5 |
|  |  |  | YES | 2 | 32 | 34 |
|  |  | Total | | 7 | 32 | 39 |
| p-value for time effect: 0.790 | |  |  |  |  |  |
| Does the facility provide the following elements of post-rape care/care to survivors of sexual violence?  b) Emergency contraception: | | **During pandemic vs. Post pandemic** | | | | |
|  |  |  |  |  |  |  |
|  |  |  | | Post | | Total |
|  |  |  |  | NO | YES |  |
|  |  | During | NO | 8 | 1 | 9 |
|  |  |  | YES | 3 | 27 | 30 |
|  |  | Total | | 11 | 28 | 39 |
| p-value for time effect: 0.913 | |  |  |  |  |  |
| Does the facility provide the following elements of post-rape care/care to survivors of sexual violence?  c) HIV post-exposure prophylaxis: | | **During pandemic vs. Post pandemic** | | | | |
|  |  |  |  |  |  |  |
|  |  |  | | Post | | Total |
|  |  |  |  | NO | YES |  |
|  |  | During | NO | 10 | 0 | 10 |
|  |  |  | YES | 0 | 29 | 29 |
|  |  | Total | | 10 | 29 | 39 |
| p-value for time effect: 0.978 | |  |  |  |  |  |
| Does the facility provide the following elements of post-rape care/care to survivors of sexual violence?  d) STI prophylaxis/presumptive treatment | | **During pandemic vs. Post pandemic** | | | | |
|  |  |  |  |  |  |  |
|  |  |  | | Post | | Total |
|  |  |  |  | NO | YES |  |
|  |  | During | NO | 12 | 2 | 14 |
|  |  |  | YES | 0 | 24 | 24 |
|  |  | Total | | 12 | 26 | 38 |
| p-value for time effect: 0.862 | |  |  |  |  |  |
| Does the facility provide the following elements of post-rape care/care to survivors of sexual violence?  e) Medical abortion to those who get pregnant: | | **During pandemic vs. Post pandemic** | | | | |
|  |  |  |  |  |  |  |
|  |  |  | | Post | | Total |
|  |  |  |  | NO | YES |  |
|  |  | During | NO | 25 | 2 | 27 |
|  |  |  | YES | 2 | 9 | 11 |
|  |  | Total | | 27 | 11 | 38 |
| p-value for time effect: 0.311 | |  |  |  |  |  |
|  | |  |  |  |  |  |
| Does the facility provide the following elements of post-rape care/care to survivors of sexual violence?  f) Surgical abortion to those who get pregnant: | | **During pandemic vs. Post pandemic** | | | | |
|  |  |  |  |  |  |  |
|  |  |  | | Post | | Total |
|  |  |  |  | NO | YES |  |
|  |  | During | NO | 25 | 2 | 27 |
|  |  |  | YES | 4 | 6 | 10 |
|  |  | Total | | 29 | 8 | 37 |
| p-value for time effect: 0.315 | |  |  |  |  |  |
| Does the facility provide the following elements of post-rape care/care to survivors of sexual violence?  g) ANC and delivery care to those who get pregnant: | | **During pandemic vs. Post pandemic** | | | | |
|  |  |  |  |  |  |  |
|  |  |  | | Post | | Total |
|  |  |  |  | NO | YES |  |
|  |  | During | NO | 10 | 0 | 10 |
|  |  |  | YES | 3 | 25 | 28 |
|  |  | Total | | 13 | 25 | 38 |
| p-value for time effect: 0.910 | |  |  |  |  |  |
| Does the facility provide the following elements of post-rape care/care to survivors of sexual violence?  h) Psychological support/crisis counselling/first line-support: | | **During pandemic vs. Post pandemic** | | | | |
|  |  |  |  |  |  |  |
|  |  |  | | Post | | Total |
|  |  |  |  | NO | YES |  |
|  |  | During | NO | 10 | 0 | 10 |
|  |  |  | YES | 3 | 26 | 29 |
|  |  | Total | | 13 | 26 | 39 |
| p-value for time effect: 0.963 | |  |  |  |  |  |
| Does the facility provide the following elements of post-rape care/care to survivors of sexual violence?  i) Referral to mental health or specialized VAW services outside the health facility: | | **During pandemic vs. Post pandemic** | | | | |
|  |  |  |  |  |  |  |
|  |  |  | | Post | | Total |
|  |  |  |  | NO | YES |  |
|  |  | During | NO | 10 | 2 | 12 |
|  |  |  | YES | 2 | 24 | 26 |
|  |  | Total | | 12 | 26 | 38 |
| p-value for time effect: 0.633 | |  |  |  |  |  |
| Do you have a referral directory with names and contact details of organizations/services that respond to cases of violence? | | **During pandemic vs. Post pandemic** | | | | |
|  |  |  |  |  |  |  |
|  |  |  | | Post | | Total |
|  |  |  |  | NO | YES |  |
|  |  | During | NO | 14 | 6 | 20 |
|  |  |  | YES | 4 | 14 | 18 |
|  |  | Total | | 18 | 20 | 38 |
| p-value for time effect: 0.674 | |  |  |  |  |  |
| The counselling rooms are separate and available for private and confidential consultation | | **During pandemic vs. Post pandemic** | | | | |
|  |  |  |  |  |  |  |
|  |  |  | | Post | | Total |
|  |  |  |  | NO | YES |  |
|  |  | During | NO | 12 | 6 | 18 |
|  |  |  | YES | 3 | 18 | 21 |
|  |  | Total | | 15 | 24 | 39 |
| p-value for time effect: 0.890 | |  |  |  |  |  |
| The examination rooms are separate and available for private and confidential consultation | | **During pandemic vs. Post pandemic** | | | | |
|  |  |  |  |  |  |  |
|  |  |  | | Post | | Total |
|  |  |  |  | NO | YES |  |
|  |  | During | NO | 13 | 4 | 17 |
|  |  |  | YES | 2 | 20 | 22 |
|  |  | Total | | 15 | 24 | 39 |
| p-value for time effect: 0.657 | |  |  |  |  |  |
| There are visible posters about violence against women (e.g. that it is not acceptable, can cause harm/health problems, you can discuss with your provider) and/or leaflets available: | | **During pandemic vs. Post pandemic** | | | | |
|  |  |  |  |  |  |  |
|  |  |  | | Post | | Total |
|  |  |  |  | NO | YES |  |
|  |  | During | NO | 19 | 7 | 26 |
|  |  |  | YES | 1 | 12 | 13 |
|  |  | Total | | 20 | 19 | 39 |
| p-value for time effect: 0.389 | |  |  |  |  |  |
| Does the facility use any of the following to maintain confidentiality of the documentation of cases of violence?  a) The facility does not have any method for maintaining confidentiality of documentation of violence | | **During pandemic vs. Post pandemic** | | | | |
|  |  |  |  |  |  |  |
|  |  |  | | Post | | Total |
|  |  |  |  | NO | YES |  |
|  |  | During | NO | 18 | 8 | 26 |
|  |  |  | YES | 7 | 5 | 12 |
|  |  | Total | | 25 | 13 | 38 |
| p-value for time effect: 0.860 | |  |  |  |  |  |
| Does the facility use any of the following to maintain confidentiality of the documentation of cases of violence?  b) Identifying information is removed or kept separate from incident record | | **During pandemic vs. Post pandemic** | | | | |
|  |  |  |  |  |  |  |
|  |  |  | | Post | | Total |
|  |  |  |  | NO | YES |  |
|  |  | During | NO | 16 | 6 | 22 |
|  |  |  | YES | 3 | 13 | 16 |
|  |  | Total | | 19 | 19 | 38 |
| p-value for time effect: 0.719 | |  |  |  |  |  |
| Does the facility use any of the following to maintain confidentiality of the documentation of cases of violence?  c) Records, registers and forms are kept in a secure storage with lock and pre-determined access | | **During pandemic vs. Post pandemic** | | | | |
|  |  |  |  |  |  |  |
|  |  |  | | Post | | Total |
|  |  |  |  | NO | YES |  |
|  |  | During | NO | 13 | 3 | 16 |
|  |  |  | YES | 4 | 18 | 22 |
|  |  | Total | | 17 | 21 | 38 |
| p-value for time effect: 0.432 | |  |  |  |  |  |
| Does the facility use any of the following to maintain confidentiality of the documentation of cases of violence?  d) Electronic medical records are password protected with pre-determined access for those who need to care for survivors | | **During pandemic vs. Post pandemic** | | | | |
|  |  |  |  |  |  |  |
|  |  |  | | Post | | Total |
|  |  |  |  | NO | YES |  |
|  |  | During | NO | 23 | 4 | 27 |
|  |  |  | YES | 2 | 7 | 9 |
|  |  | Total | | 25 | 11 | 36 |
| p-value for time effect: 0.265 | |  |  |  |  |  |
| Does the facility use any of the following to maintain confidentiality of the documentation of cases of violence?  e) Any take home cards or information does not have any direct indication of the survivor’s abuse | | **During pandemic vs. Post pandemic** | | | | |
|  |  |  |  |  |  |  |
|  |  |  | | Post | | Total |
|  |  |  |  | NO | YES |  |
|  |  | During | NO | 23 | 5 | 28 |
|  |  |  | YES | 2 | 8 | 10 |
|  |  | Total | | 25 | 13 | 38 |
| p-value for time effect: 0.501 | |  |  |  |  |  |
| Does the facility use any of the following to maintain confidentiality of the documentation of cases of violence?  f) Other | | **During pandemic vs. Post pandemic** | | | | |
|  |  |  |  |  |  |  |
|  |  |  | | Post | | Total |
|  |  |  |  | NO | YES |  |
|  |  | During | NO | 26 | 1 | 27 |
|  |  |  | YES | 2 | 1 | 3 |
|  |  | Total | | 28 | 2 | 30 |
| p-value for time effect: 0.787 | |  |  |  |  |  |
| Does the facility have the following essential supplies and equipment for post-rape care today during this visit?  a) Emergency contraception | | **During pandemic vs. Post pandemic** | | | | |
|  |  |  |  |  |  |  |
|  |  |  | | Post | | Total |
|  |  |  |  | NO | YES |  |
|  |  | During | NO | 9 | 4 | 13 |
|  |  |  | YES | 1 | 25 | 26 |
|  |  | Total | | 10 | 29 | 39 |
| p-value for time effect: 0.695 | |  |  |  |  |  |
| Does the facility have the following essential supplies and equipment for post-rape care today during this visit?  b) Antiretroviral drugs for post-exposure prophylaxis for HIV prevention | | **During pandemic vs. Post pandemic** | | | | |
|  |  |  |  |  |  |  |
|  |  |  | | Post | | Total |
|  |  |  |  | NO | YES |  |
|  |  | During | NO | 10 | 1 | 11 |
|  |  |  | YES | 2 | 26 | 28 |
|  |  | Total | | 12 | 27 | 39 |
| p-value for time effect: 0.342 | |  |  |  |  |  |
| Does the facility have the following essential supplies and equipment for post-rape care today during this visit?  c) Antibiotics/other drugs for treatment or prophylaxis for sexually transmitted infection | | **During pandemic vs. Post pandemic** | | | | |
|  |  |  |  |  |  |  |
|  |  |  | | Post | | Total |
|  |  |  |  | NO | YES |  |
|  |  | During | NO | 10 | 1 | 11 |
|  |  |  | YES | 3 | 25 | 28 |
|  |  | Total | | 13 | 26 | 39 |
| p-value for time effect: 0.351 | |  |  |  |  |  |
| Does the facility have the following essential supplies and equipment for post-rape care today during this visit?  d) Hepatitis B vaccination | | **During pandemic vs. Post pandemic** | | | | |
|  |  |  |  |  |  |  |
|  |  |  | | Post | | Total |
|  |  |  |  | NO | YES |  |
|  |  | During | NO | 18 | 5 | 23 |
|  |  |  | YES | 2 | 14 | 16 |
|  |  | Total | | 20 | 19 | 39 |
| p-value for time effect: 0.695 | |  |  |  |  |  |
| Does the facility have the following essential supplies and equipment for post-rape care today during this visit?  e) Job aids (for example, flow charts, algorithms, pictograms) | | **During pandemic vs. Post pandemic** | | | | |
|  |  |  |  |  |  |  |
|  |  |  | | Post | | Total |
|  |  |  |  | NO | YES |  |
|  |  | During | NO | 13 | 8 | 21 |
|  |  |  | YES | 3 | 15 | 18 |
|  |  | Total | | 16 | 23 | 39 |
| p-value for time effect: 0.329 | |  |  |  |  |  |
| Does the facility have the following essential supplies and equipment for post-rape care today during this visit?  f) Documentation forms (for example, medical intake forms, police forms for forensic evidence, medico-kegal certificates, referral forms) | | **During pandemic vs. Post pandemic** | | | | |
|  |  |  |  |  |  |  |
|  |  |  | | Post | | Total |
|  |  |  |  | NO | YES |  |
|  |  | During | NO | 12 | 4 | 16 |
|  |  |  | YES | 3 | 20 | 23 |
|  |  | Total | | 15 | 24 | 39 |
| p-value for time effect: 0.623 | |  |  |  |  |  |
| Does the facility have the following essential supplies and equipment for post-rape care today during this visit?  g) Communication materials about violence against women | | **During pandemic vs. Post pandemic** | | | | |
|  |  |  |  |  |  |  |
|  |  |  | | Post | | Total |
|  |  |  |  | NO | YES |  |
|  |  | During | NO | 15 | 11 | 26 |
|  |  |  | YES | 0 | 13 | 13 |
|  |  | Total | | 15 | 24 | 39 |
| p-value for time effect: 0.471 | |  |  |  |  |  |
| Does the facility have the following essential supplies and equipment for post-rape care today during this visit?  h) Sanitary pads | | **During pandemic vs. Post pandemic** | | | | |
|  |  |  |  |  |  |  |
|  |  |  | | Post | | Total |
|  |  |  |  | NO | YES |  |
|  |  | During | NO | 15 | 6 | 21 |
|  |  |  | YES | 4 | 14 | 18 |
|  |  | Total | | 19 | 20 | 39 |
| p-value for time effect: 0.820 | |  |  |  |  |  |
| Does the facility have the following essential supplies and equipment for post-rape care today during this visit?  i) Examination table/couch (with curtains or screens if needed for privacy) | | **During pandemic vs. Post pandemic** | | | | |
|  |  |  |  |  |  |  |
|  |  |  | | Post | | Total |
|  |  |  |  | NO | YES |  |
|  |  | During | NO | 10 | 2 | 12 |
|  |  |  | YES | 1 | 26 | 27 |
|  |  | Total | | 11 | 28 | 39 |
| p-value for time effect: <0.001 | |  |  |  |  |  |
| Does the facility have the following essential supplies and equipment for post-rape care today during this visit?  j) Secure record storage cabinets with a lock | | **During pandemic vs. Post pandemic** | | | | |
|  |  |  |  |  |  |  |
|  |  |  | | Post | | Total |
|  |  |  |  | NO | YES |  |
|  |  | During | NO | 15 | 5 | 20 |
|  |  |  | YES | 1 | 18 | 19 |
|  |  | Total | | 16 | 23 | 39 |
| p-value for time effect: 0.385 | |  |  |  |  |  |
| Does the facility have the following essential supplies and equipment for post-rape care today during this visit?  k) Adequate light source (lamp or torch) | | **During pandemic vs. Post pandemic** | | | | |
|  |  |  |  |  |  |  |
|  |  |  | | Post | | Total |
|  |  |  |  | NO | YES |  |
|  |  | During | NO | 7 | 0 | 7 |
|  |  |  | YES | 2 | 30 | 32 |
|  |  | Total | | 9 | 30 | 39 |
| p-value for time effect: 0.938 | |  |  |  |  |  |
| Does the facility have the following essential supplies and equipment for post-rape care today during this visit?  l) Speculum | | **During pandemic vs. Post pandemic** | | | | |
|  |  |  |  |  |  |  |
|  |  |  | | Post | | Total |
|  |  |  |  | NO | YES |  |
|  |  | During | NO | 8 | 1 | 9 |
|  |  |  | YES | 1 | 29 | 30 |
|  |  | Total | | 9 | 30 | 39 |
| p-value for time effect: 0.782 | |  |  |  |  |  |
| Does the facility have the following essential supplies and equipment for post-rape care today during this visit?  m) Pregnancy testing kits | | **During pandemic vs. Post pandemic** | | | | |
|  |  |  |  |  |  |  |
|  |  |  | | Post | | Total |
|  |  |  |  | NO | YES |  |
|  |  | During | NO | 8 | 5 | 13 |
|  |  |  | YES | 0 | 26 | 26 |
|  |  | Total | | 8 | 31 | 39 |
| p-value for time effect: 0.844 | |  |  |  |  |  |
| Does the facility have the following essential supplies and equipment for post-rape care today during this visit?  n) Rapid tests for HIV/syphilis | | **During pandemic vs. Post pandemic** | | | | |
|  |  |  |  |  |  |  |
|  |  |  | | Post | | Total |
|  |  |  |  | NO | YES |  |
|  |  | During | NO | 11 | 4 | 15 |
|  |  |  | YES | 0 | 24 | 24 |
|  |  | Total | | 11 | 28 | 39 |
| p-value for time effect: 0.572 | |  |  |  |  |  |
| Does the facility have the following essential supplies and equipment for post-rape care today during this visit?  o) Urinalysis kits | | **During pandemic vs. Post pandemic** | | | | |
|  |  |  |  |  |  |  |
|  |  |  | | Post | | Total |
|  |  |  |  | NO | YES |  |
|  |  | During | NO | 11 | 5 | 16 |
|  |  |  | YES | 1 | 21 | 22 |
|  |  | Total | | 12 | 26 | 38 |
| p-value for time effect: 0.974 | |  |  |  |  |  |
| Does the facility have the following essential supplies and equipment for post-rape care today during this visit?  p) Test strips for vaginal infections | | **During pandemic vs. Post pandemic** | | | | |
|  |  |  |  |  |  |  |
|  |  |  | | Post | | Total |
|  |  |  |  | NO | YES |  |
|  |  | During | NO | 13 | 6 | 19 |
|  |  |  | YES | 4 | 16 | 20 |
|  |  | Total | | 17 | 22 | 39 |
| p-value for time effect: 0.423 | |  |  |  |  |  |
| Does the facility have the following essential supplies and equipment for post-rape care today during this visit?  q) Supplies for wound care | | **During pandemic vs. Post pandemic** | | | | |
|  |  |  |  |  |  |  |
|  |  |  | | Post | | Total |
|  |  |  |  | NO | YES |  |
|  |  | During | NO | 9 | 1 | 10 |
|  |  |  | YES | 3 | 26 | 29 |
|  |  | Total | | 12 | 27 | 39 |
| p-value for time effect: 0.372 | |  |  |  |  |  |
| Does the facility have the following essential supplies and equipment for post-rape care today during this visit?  r) Analgesics | | **During pandemic vs. Post pandemic** | | | | |
|  |  |  |  |  |  |  |
|  |  |  | | Post | | Total |
|  |  |  |  | NO | YES |  |
|  |  | During | NO | 9 | 0 | 9 |
|  |  |  | YES | 2 | 28 | 30 |
|  |  | Total | | 11 | 28 | 39 |
| p-value for time effect: 0.369 | |  |  |  |  |  |
| Does the facility have the following essential supplies and equipment for post-rape care today during this visit?  s) Anti-emetics | | **During pandemic vs. Post pandemic** | | | | |
|  |  |  |  |  |  |  |
|  |  |  | | Post | | Total |
|  |  |  |  | NO | YES |  |
|  |  | During | NO | 11 | 2 | 13 |
|  |  |  | YES | 2 | 24 | 26 |
|  |  | Total | | 13 | 26 | 39 |
| p-value for time effect: 0.495 | |  |  |  |  |  |
| Does the facility have the following essential supplies and equipment for post-rape care today during this visit?  t) Tetanus toxoid | | **During pandemic vs. Post pandemic** | | | | |
|  |  |  |  |  |  |  |
|  |  |  | | Post | | Total |
|  |  |  |  | NO | YES |  |
|  |  | During | NO | 9 | 4 | 13 |
|  |  |  | YES | 1 | 25 | 26 |
|  |  | Total | | 10 | 29 | 39 |
| p-value for time effect: <0.001 | |  |  |  |  |  |
| Does the facility have the following essential supplies and equipment for post-rape care today during this visit?  u) Consent forms | | **During pandemic vs. Post pandemic** | | | | |
|  |  |  |  |  |  |  |
|  |  |  | | Post | | Total |
|  |  |  |  | NO | YES |  |
|  |  | During | NO | 10 | 10 | 20 |
|  |  |  | YES | 3 | 16 | 19 |
|  |  | Total | | 13 | 26 | 39 |
| p-value for time effect: 0.650 | |  |  |  |  |  |
| Does the facility have the following essential supplies and equipment for post-rape care today during this visit?  v) Sheets, blankets, and towels | | **During pandemic vs. Post pandemic** | | | | |
|  |  |  |  |  |  |  |
|  |  |  | | Post | | Total |
|  |  |  |  | NO | YES |  |
|  |  | During | NO | 12 | 9 | 21 |
|  |  |  | YES | 3 | 15 | 18 |
|  |  | Total | | 15 | 24 | 39 |
| p-value for time effect: 0.703 | |  |  |  |  |  |
| Have health care providers received training on responding to intimate partner/domestic violence in the last 6 months? | | **During pandemic vs. Post pandemic** | | | | |
|  |  |  |  |  |  |  |
|  |  |  | | Post | | Total |
|  |  |  |  | NO | YES |  |
|  |  | During | NO | 20 | 4 | 24 |
|  |  |  | YES | 7 | 8 | 15 |
|  |  | Total | | 27 | 12 | 39 |
| p-value for time effect: <0.001 | |  |  |  |  |  |
| Have health care providers received training on responding to sexual violence in the last 6 months? | | **During pandemic vs. Post pandemic** | | | | |
|  |  |  |  |  |  |  |
|  |  |  | | Post | | Total |
|  |  |  |  | NO | YES |  |
|  |  | During | NO | 22 | 4 | 26 |
|  |  |  | YES | 5 | 8 | 13 |
|  |  | Total | | 27 | 12 | 39 |
| p-value for time effect: 0.692 | |  |  |  |  |  |
|  | |  |  |  |  |  |

*Effect of time (endline vs. baseline) in key variables of Family Planning form.*

*A multilevel longitudinal logistic model, with "country" as a random effect, was used to assess significance of relevant effects (p values).*

**Supplementary table A5: STI and HIV SERVICES**

| CROSSTABULATIONS (post pandemic] vs. during pandemic) | | | | | |
| --- | --- | --- | --- | --- | --- |
|  |  |  |  |  |  |
| Does this facility offer HIV counselling and testing services | **During pandemic vs. Post pandemic** | | | | |
|  |  |  |  |  |  |
|  |  | | Post | | Total |
|  |  |  | NO | YES |  |
|  | During | NO | 4 | 0 | 4 |
|  |  | YES | 1 | 34 | 35 |
|  | Total | | 5 | 34 | 39 |
| p-value for time effect: 0.501 |  |  |  |  |  |
| Do you have the national HIV counselling and testing guidelines available in this facility today? | **During pandemic vs. Post pandemic** | | | | |
|  |  |  |  |  |  |
|  |  | | Post | | Total |
|  |  |  | NO | YES |  |
|  | During | NO | 5 | 3 | 8 |
|  |  | YES | 0 | 31 | 31 |
|  | Total | | 5 | 34 | 39 |
| p-value for time effect: 0.474 |  |  |  |  |  |
| Does this facility have HIV rapid test kits (with valid expiration date) in stock today, ready for client testing? | **During pandemic vs. Post pandemic** | | | | |
|  |  |  |  |  |  |
|  |  | | Post | | Total |
|  |  |  | NO | YES |  |
|  | During | NO | 6 | 3 | 9 |
|  |  | YES | 0 | 30 | 30 |
|  | Total | | 6 | 33 | 39 |
| p-value for time effect: 0.968 |  |  |  |  |  |
| Does this facility have condoms available in this service site today to give to clients receiving services? | **During pandemic vs. Post pandemic** | | | | |
|  |  |  |  |  |  |
|  |  | | Post | | Total |
|  |  |  | NO | YES |  |
|  | During | NO | 8 | 3 | 11 |
|  |  | YES | 2 | 26 | 28 |
|  | Total | | 10 | 29 | 39 |
| p-value for time effect: 0.362 |  |  |  |  |  |
| Does this facility offer any of the following tests on-site?  Syphilis Rapid Testing | **During pandemic vs. Post pandemic** | | | | |
|  |  |  |  |  |  |
|  |  | | Post | | Total |
|  |  |  | NO | YES |  |
|  | During | NO | 5 | 3 | 8 |
|  |  | YES | 1 | 26 | 27 |
|  | Total | | 6 | 29 | 35 |
| p-value for time effect: 0.583 |  |  |  |  |  |
| Does this facility offer any of the following tests on-site?  HIV rapid testing | **During pandemic vs. Post pandemic** | | | | |
|  |  |  |  |  |  |
|  |  | | Post | | Total |
|  |  |  | NO | YES |  |
|  | During | NO | 3 | 0 | 3 |
|  |  | YES | 1 | 32 | 33 |
|  | Total | | 4 | 32 | 36 |
| p-value for time effect: 0.995 |  |  |  |  |  |
|  |  |  |  |  |  |
| Does this facility offer any of the following tests on-site?  Urine rapid tests for pregnancy | **During pandemic vs. Post pandemic** | | | | |
|  |  |  |  |  |  |
|  |  | | Post | | Total |
|  |  |  | NO | YES |  |
|  | During | NO | 1 | 2 | 3 |
|  |  | YES | 3 | 30 | 33 |
|  | Total | | 4 | 32 | 36 |
| p-value for time effect: 0.046 |  |  |  |  |  |
| Does this facility offer any of the following tests on-site?  Dry Blood Spot (DBS) collection for HIV viral load or EID | **During pandemic vs. Post pandemic** | | | | |
|  |  |  |  |  |  |
|  |  | | Post | | Total |
|  |  |  | NO | YES |  |
|  | During | NO | 9 | 5 | 14 |
|  |  | YES | 5 | 17 | 22 |
|  | Total | | 14 | 22 | 36 |
| p-value for time effect: 0.489 |  |  |  |  |  |
| Do providers in this facility diagnose STIs? | **During pandemic vs. Post pandemic** | | | | |
|  |  |  |  |  |  |
|  |  | | Post | | Total |
|  |  |  | NO | YES |  |
|  | During | NO | 0 | 0 | 0 |
|  |  | YES | 0 | 39 | 39 |
|  | Total | | 0 | 39 | 39 |
| p-value for time effect: na | | | | | |
| Do you have the national guidelines for the diagnosis and treatment of STIs available in this facility today? | **During pandemic vs. Post pandemic** | | | | |
|  |  |  |  |  |  |
|  |  | | Post | | Total |
|  |  |  | NO | YES |  |
|  | During | NO | 4 | 2 | 6 |
|  |  | YES | 1 | 32 | 33 |
|  | Total | | 5 | 34 | 39 |
| p-value for time effect: 0.325 |  |  |  |  |  |
| Have you or any provider(s) of STI services received any training in STI diagnosis and treatment in the last two years? | **During pandemic vs. Post pandemic** | | | | |
|  |  |  |  |  |  |
|  |  | | Post | | Total |
|  |  |  | NO | YES |  |
|  | During | NO | 11 | 9 | 20 |
|  |  | YES | 1 | 18 | 19 |
|  | Total | | 12 | 27 | 39 |
| p-value for time effect: 0.535 |  |  |  |  |  |
| Does this facility or other public healthcare facilities in your area offer immunization services? | **During pandemic vs. Post pandemic** | | | | |
|  |  |  |  |  |  |
|  |  | | Post | | Total |
|  |  |  | NO | YES |  |
|  | During | NO | 8 | 1 | 9 |
|  |  | YES | 2 | 27 | 29 |
|  | Total | | 10 | 28 | 38 |
| p-value for time effect: 0.899 |  |  |  |  |  |
| Is the HIV and STIs testing and counselling service room or area a private room/area with auditory and visual privacy? | **During pandemic vs. Post pandemic** | | | | |
|  |  |  |  |  |  |
|  |  | | Post | | Total |
|  |  |  | NO | YES |  |
|  | During | NO | 1 | 3 | 4 |
|  |  | YES | 1 | 21 | 22 |
|  | Total | | 2 | 24 | 26 |
| p-value for time effect: 0.579 |  |  |  |  |  |
| Do you have guidelines for partner notification and contact tracing available? Partner Notification (PN) (for HIV) yes, national guidelines | **During pandemic vs. Post pandemic** | | | | |
|  |  |  |  |  |  |
|  |  | | Post | | Total |
|  |  |  | NO | YES |  |
|  | During | NO | 5 | 4 | 9 |
|  |  | YES | 0 | 29 | 29 |
|  | Total | | 5 | 33 | 38 |
| p-value for time effect: 0.369 |  |  |  |  |  |
| Do you have guidelines for partner notification and contact tracing available? (for HIV) yes, local guidelines | **During pandemic vs. Post pandemic** | | | | |
|  |  |  |  |  |  |
|  |  | | Post | | Total |
|  |  |  | NO | YES |  |
|  | During | NO | 14 | 4 | 18 |
|  |  | YES | 3 | 12 | 15 |
|  | Total | | 17 | 16 | 33 |
| p-value for time effect: 0.541 |  |  |  |  |  |
| Do you have guidelines for partner notification and contact tracing available? (for other STIs) yes, national guidelines | **During pandemic vs. Post pandemic** | | | | |
|  |  |  |  |  |  |
|  |  | | Post | | Total |
|  |  |  | NO | YES |  |
|  | During | NO | 8 | 4 | 12 |
|  |  | YES | 0 | 23 | 23 |
|  | Total | | 8 | 27 | 35 |
| p-value for time effect: 0.738 |  |  |  |  |  |
| Do you have guidelines for partner notification and contact tracing available? (for other STIs) yes, local guidelines | **During pandemic vs. Post pandemic** | | | | |
|  |  |  |  |  |  |
|  |  | | Post | | Total |
|  |  |  | NO | YES |  |
|  | During | NO | 14 | 5 | 19 |
|  |  | YES | 3 | 12 | 15 |
|  | Total | | 17 | 17 | 34 |
| p-value for time effect: 0.658 |  |  |  |  |  |
| Do you have guidelines for partner notification and contact tracing available? Contact Tracing (CT) (for HIV) yes, national guidelines | **During pandemic vs. Post pandemic** | | | | |
|  |  |  |  |  |  |
|  |  | | Post | | Total |
|  |  |  | NO | YES |  |
|  | During | NO | 7 | 4 | 11 |
|  |  | YES | 4 | 21 | 25 |
|  | Total | | 11 | 25 | 36 |
| p-value for time effect: 0.646 |  |  |  |  |  |
| Do you have guidelines for partner notification and contact tracing available? (for HIV) yes, local guidelines | **During pandemic vs. Post pandemic** | | | | |
|  |  |  |  |  |  |
|  |  | | Post | | Total |
|  |  |  | NO | YES |  |
|  | During | NO | 13 | 5 | 18 |
|  |  | YES | 4 | 12 | 16 |
|  | Total | | 17 | 17 | 34 |
| p-value for time effect: 0.541 |  |  |  |  |  |
| Do you have guidelines for partner notification and contact tracing available? (for other STIs) yes, national guidelines | **During pandemic vs. Post pandemic** | | | | |
|  |  |  |  |  |  |
|  |  | | Post | | Total |
|  |  |  | NO | YES |  |
|  | During | NO | 9 | 4 | 13 |
|  |  | YES | 3 | 20 | 23 |
|  | Total | | 12 | 24 | 36 |
| p-value for time effect: 0.936 |  |  |  |  |  |
| Do you have guidelines for partner notification and contact tracing available? (for other STIs) yes, local guidelines | **During pandemic vs. Post pandemic** | | | | |
|  |  |  |  |  |  |
|  |  | | Post | | Total |
|  |  |  | NO | YES |  |
|  | During | NO | 13 | 4 | 17 |
|  |  | YES | 5 | 12 | 17 |
|  | Total | | 18 | 16 | 34 |
| p-value for time effect: 0.486 |  |  |  |  |  |
|  |  |  |  |  |  |

*Effect of time (endline vs. baseline) in key variables of Family Planning form.*

*A multilevel longitudinal logistic model, with "country" as a random effect, was used to assess significance of relevant effects (p values).*
